# Fungal Contaminants in Energy Efficient Dwellings: Impact of Ventilation Type and Level of Urbanization

**DOI:** 10.3390/ijerph17144936

**Published:** 2020-07-08

**Authors:** Hélène Niculita-Hirzel, Shen Yang, Corinne Hager Jörin, Vincent Perret, Dusan Licina, Joëlle Goyette Pernot

**Affiliations:** 1 Department of Occupational Health and Environment, Center for Primary Care and Public Health (Unisanté), University of Lausanne, CH-1066 Epalinges, Switzerland; 2 Human-Oriented Built Environment Lab, School of Architecture, Civil and Environmental Engineering, École Polytechnique Fédérale de Lausanne, CH-1015 Lausanne, Switzerland; shen.yang@epfl.ch (S.Y.); dusan.licina@epfl.ch (D.L.); 3 HumanTech Institute, School of Engineering and Architecture of Fribourg, HES-SO University of Applied Sciences and Arts Western Switzerland, CH-1700 Fribourg, Switzerland; Corinne.HagerJoerin@hefr.ch; 4 TOXpro SA, CH-1227 Geneva, Switzerland; vincent.perret@toxpro.ch; 5 Transform Institute, School of Engineering and Architecture of Fribourg, HES-SO University of Applied Sciences and Arts Western Switzerland, CH-1700 Fribourg, Switzerland; Joelle.Goyette@hefr.ch

**Keywords:** home environment, visible moulds, ventilation type, geographic location, settled dust

## Abstract

The presence of growing fungi in the indoor environment has been associated with the development of respiratory problems such as asthma or allergic rhinitis, as well as the worsening of respiratory pathologies. Their proliferation indoors could be a result of water leakage or inadequate ventilation. Although the factors promoting mould growth have been widely investigated in traditional dwellings, little work has been done in energy efficient dwellings. Here, the effectiveness of ventilation type, i.e., natural or mechanical, in influencing mould development was estimated in 44 recent and 105 retrofitted energy efficient dwellings. Fungi growing on surfaces were investigated in the dwellings situated in rural, peri-urban, and urban regions of Switzerland. The presence of these fungi was also investigated in bedroom settled dust. Information on building characteristics and owners’ lifestyle were collected. Significant associations were found with the level of urbanisation, the location of mouldy area in dwellings, and the diversity of fungal taxa. Dwellings in peri-urban zones showed the most frequent fungal contamination in the owners’ bedroom and the highest diversity of fungal genera among dwellings. While the urbanisation level or the ventilation type favoured no specific genus, we found marked disparities in the diversity of fungi growing on surfaces in naturally ventilated versus mechanically ventilated dwellings. *Aspergillus*, in particular, was a frequent surface contaminant in bedrooms with natural ventilation, but not in those mechanically ventilated. We observed a strong association between fungal growth on surfaces and the number of fungal particles counted in the settled dust of owners’ bedrooms. These results demonstrate the importance of ventilation systems in energy efficient dwellings in controlling fungal proliferation in living areas.

## 1. Introduction

The building sector has been identified as one of the key sectors to decrease energy consumption and CO_2_ emissions in developed countries [1]. Following the introduction of energy efficiency requirements in European building codes [2,3,4], all newly constructed buildings from 2021 will aim to consume nearly zero energy [5]. In addition, an energy efficient renovation program for existing buildings was introduced in the last ten years by EU policies [6]. In Switzerland particularly, a building energy certification scheme, named Minergie, was established to attest the high energy efficiency of such buildings and human comfort [7]. Additionally, an energy efficient renovation program, the *Programme Bâtiment*, was launched by the government [8]. The first requirement for the latter program is airtightness. Multiple studies have shown that increased airtightness can lead to an accumulation of indoor air pollutants if the windows are not opened regularly [9,10], which may ultimately pose adverse health effects [11]. Air tightening of dwellings may, in particular, reduce the rate of removal of the allergens and promote moisture accumulation through human activities (e.g., showering, cooking, drying laundry) [12]. High moisture levels are associated with fungal growth, which can lead to an increased risk of asthma [13]. As a control measure, mechanical ventilation in dwellings has been associated to an absence of visible moulds [14]. Yet, no large-scale comparison of the effectiveness of mechanical ventilation on fungal contamination of energy efficient dwellings exist to date. To better understand the role of residential ventilation in controlling the indoor fungal contamination, it is valuable to conduct a large field investigation in dwellings equipped with both mechanical and natural ventilation.

Fungal growth on indoor surfaces has been associated with health problems in dwelling occupants [15] through the aerosolisation of fungal particles [16]. Indeed, spores, hyphal fragments and microbial volatile organic compounds (mVOCs) can be emitted from mouldy surfaces [17] and the size of these particles is small enough to be inhaled [16,18,19]. A chronic exposure to moulds can induce a hypersensitivity in healthy adults [20], as well as respiratory problems such as asthma in young children [21,22,23], adults [24] and older adults [25]. The most common indoor fungi that can cause such pathologies are *Penicillium* spp. and *Aspergillus* spp. [17]. However, the impact of such exposure on occupants’ respiratory health is related to the location of the growing fungus in home. Significant associations with asthma and wheezing were reported for moisture damage with or without visible fungal growth in the main living areas, bedrooms [26,27,28] and kitchens [26,27] of traditional dwellings. Nevertheless, no consistent associations were found when the moulds were present in bathrooms or when their location in the house was not specified [27,28]. Furthermore, the risk of fungal exposures might increase in rural compared to urban dwellings, as the higher proportion of houses contaminated with moulds was observed in rural compared to urban areas [14]. In energy-efficient dwellings, association with asthma has been also recently reported [13].

To develop on indoor surfaces, fungi need to find favorable conditions for their growth. The most important determinants include type of building material [29], air temperature and moisture levels [30]. These three are influenced by factors such as dwelling age [31,32,33,34,35], heating/ventilation system [32,33], insulation level [36], solar irradiance [37], and occupancy density [37]. Occupants’ behavior is another driver, for example, through the altered frequency of window opening, the use of exhaust fans [32,35] or of humidifiers [35,38]. By eliminating cold bridges, it is possible to lower the risk of visible fungal growth [13]. Retrofitted dwellings with condensation issues were found to increase the risk of surfaces contamination by *Aspergillus/Penicillium* [39]. At present, limited information is available on fungal contamination in mechanically ventilated energy-efficient dwellings and on the dispersion of fungal propagules from mouldy surfaces to living area, in particular to the place where we spend most of our time, bedrooms. Furthermore, no studies have explored the importance of ventilation type and urbanization level of dwellings on the development of visible moulds. To understand the impact on occupants’ health and wellbeing, there is a need to improve the understanding of mould presence and location in energy-efficient homes with or without mechanical ventilation.

The main objectives of this study were (1) to produce a large dataset on moulds contamination of energy efficient buildings in Switzerland and (2) to investigate the relation of moulds to the level of urbanization, and to the type of ventilation (mechanical vs. natural) as well as to the dwelling condition (retrofitted vs. recently built).

## 2. Materials and Methods

### 2.1. Building Selection

The buildings investigated in ‘Mesqualair’ New Regional Policy collaborative project [40,41,42] were selected from high-energy-performance buildings registered with the Minergie Agency and Romand Cantonal Energy Service Offices. Within the full project framework, sample population that agreed to participate in our study included the owners of 433 retrofitted buildings that met the requirements of the national energy renovation project for buildings (*Programme Bâtiment;* PB) and the owners of 217 recently constructed buildings that met the requirements of Minergie (M) green building label. The homes were distributed in South-Western part of Switzerland and were assigned to rural, peri-urban or urban areas according to the national classification of the communes to which they belong [43]. A self-administered questionnaire was used to collect information about dwelling characteristics, energy consumption, ventilation system, occupant behaviour and lifestyle, as well as occupants’ satisfaction, as reported in details in our related study [41]. In summary, dwellings were single-family houses (95%) occupied by direct owners. Almost half of the owners owned pets and 9% smoked inside the dwellings. The M dwellings were built between 2001 and 2012, while the PB dwellings were built between 1650 and 1995. As many as 98% of M dwellings had a mechanical ventilation. In contrast, 96% of PB were naturally ventilated. The median values of annual electricity consumption were similar between M and PB dwellings (37 kWh/m^2^ per year for recently built M dwellings, 45 kWh/m^2^ per year for PB). Space heating of M dwellings was mainly supplied from renewable and low-carbon sources (heat pump (30%), geothermal (20%), wood pellet (18%) and solar thermal (10%)), while 40% of PB dwellings used fossil fuels for space heating. A part of the questionnaire concerned the reporting of visible mould, mouldy odour, or signs of other moisture problems. In 2014, the owners reported visible moulds in 18% of energy efficient dwellings.

### 2.2. Study Design

**Figure 1:**
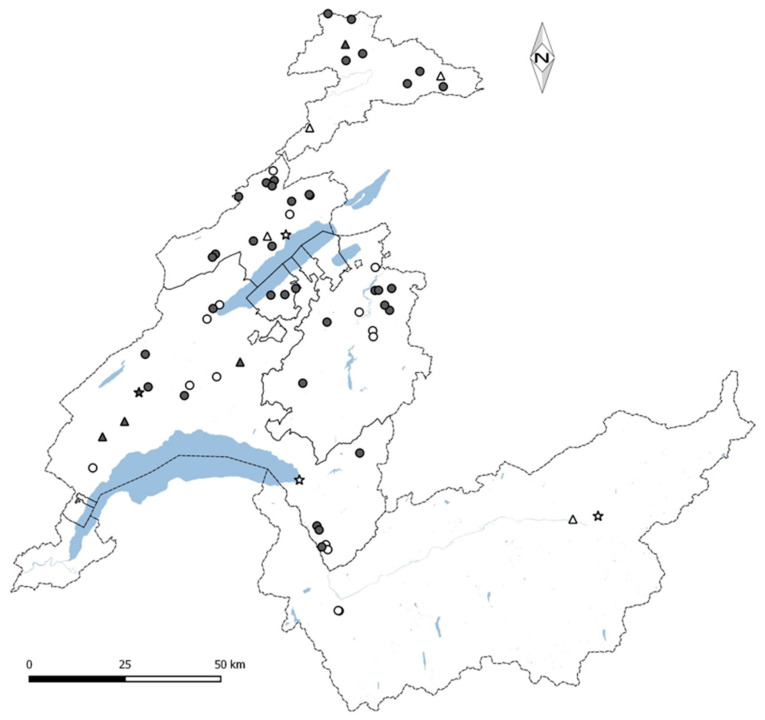
Dwellings location in South-Western part of Switzerland. The triangles indicate the Minergie dwellings with mechanical ventilation, the circles indicate the renovated dwellings with natural ventilation and the stars depict the renovated dwellings with mechanical ventilation. Dwellings with visible moulds are marked with black coloured symbols. The lakes are coloured in blue and the cantonal borders are geographic landmarks.

In 2015, we proposed to 200 out of 650 dwelling owners to participate in the current study on moulds contamination. The selection was equally split on 100 dwellings whose owners reported visible moulds the previous year and 100 owners that did not observed any. 85 and 84 owners respectively accepted our invitation; the location of their dwellings is shows in Figure 1. Out of 169 dwellings, 44 were recently built Minergie dwellings (M) and 105 were retrofitted energy-efficient dwellings (PB). Mechanical ventilation was installed in all Minergie dwellings and in only five PB dwellings. Each dwelling was investigated during the heating season 2015–2016 (with operation of the heating system from early November to late February). Each participant received by mail one kit for three surface samplings and two kits for passive settled dust sampling. The latter two kits included one electrostatic dust fall collector (EDC) and one electrostatic wipe cloth. The participants were instructed to make a single sample from each surface with visible fungal growth, to collect the settled dust on elevated surfaces of their bedrooms with the electrostatic wipe cloth and to place the EDC in their bedroom upon mail reception. After one week, they returned the surface samples (used or not) and the cloth by mail, and, after eleven additional weeks, the EDC. Out of the 169 participants, 149 returned all the samples. Written informed consent was obtained from all participants.

### 2.3. Sampling

To identify fungi growing on surfaces within dwellings, surface sampling was performed by the owner from contaminated surfaces located in all living spaces (main bedroom, living room, kitchen, bathroom, hallway, utility room). One sample was collected from each contaminated site by the tape technique method. The instructions given included several steps: (i) remove the 3 cm piece of adhesive tape (3M Scotch^®^, Lausanne, Switzerland) provided onto the glass microscope slide (Thermo Scientific™ SuperFrost Ultra Plus™ GOLD Adhesion Slides© 2020 Thermo Fisher Scientific Inc. provided by Fisher Scientific AG, Reinach, Switerzerland) and press firmly the adhesive side over the contaminated area; (ii) remove the tape from the surface and label the location of the sample. The collected samples were placed into the provided box and sent to the lab by mail.

The owners were also instructed to sample the settled dust in his/her bedroom by two passive methods: With a cloth and with EDC. The first method consisted of settled-dust collection from 1 m^2^ surface of furniture well above floor level with the provided electrostatic wipe cloth (Swiffer^®^ Sweeper dry, provided by Coop, Lausanne, Switzerland). This model has already been used in previous studies [44]. After sampling, the cloth was placed in a zip lock re-sealable plastic bag which was previously labelled. The second method consisted of passive dust collection between 1.20 and 1.60 m above the floor over a period of twelve weeks via the electrostatic properties of the wipe (Swiffer^®^ Sweeper dry) fixed into a 19 × 28 cm polypropylene box with white glue (UHU^®^, Bühl, Germany). The use of such EDCs was first reported by Noss et al. [45] and was previously validated for its efficiency to trap the overall fungal diversity present in aerosols [46]. After twelve weeks, the EDC was closed, and the date and sampling location was written on its cover. Then, the EDC was sealed in labelled plastic bags and sent to the lab by mail.

### 2.4. Culture

The surface samples were firstly analysed by direct microscopic examination. After that, the adhesive tape was removed from the support, cut into two pieces with the adhesive side placed downwards on a plate with potato dextrose agar (PDA) medium (Oxoid^®^, Basingstoke, England) and the other piece placed on a malt extract agar (MEA) plate (Oxoid^®^). Then, the plates were incubated at 25 °C for further identification.

Each wipe (cloth and EDC) was put in a plastic bag with a washing solution of 20 mL of sterile 0.1% Tween 80 solution (Merck^®^, Darmstadt, Germany) and shaken for ten minutes in a Stomacher™ (AES^®^, Combourg, France) [47]. 10 ± 0.5 mL of the washing solution was then collected. A serial dilution of 100 μL of the harvested suspension were spread in replicates on plates with dichloran-glycerol culture medium (DG18) (Oxoid^®^) and placed in an incubator at 25 °C for five days. All plates were checked daily for fungal colony growth and fungal colony forming units (CFUs) were numbered on each Petri dish. Representative colonies were isolated and cultured on PDA and MEA plates for further identification.

### 2.5. Identification of Fungi

All PDA and MEA plates were incubated for 5–7 days at 25 °C. Each colony was identified at the genus level by macroscopic observation of the growth characteristics of the colonies and microscopic observation of conidiophores, branching patterns of conidiophores and conidiogenous cells morphology [48,49,50,51].

### 2.6. Statistical Analysis

The non-parametric Mann–Whitney U test was performed to compare outcomes between two independent groups (e.g., CFUs number between mechanically and naturally ventilated dwellings, or between Minergie and Program Bâtiment dwelling types). Chi-square test was applied to examine the statistical differences among different categories (e.g., incidences of occupants’ self-reported health issues and dwelling location or ventilation type). Binary logistic regression was applied for calculation of odds ratios. The statistical analyses were performed using STATA 15.0 (StataCorp LLC, College Station, TX, USA) and SPSS 14.0 (SPSS Inc., Chicago, IL, USA) software.

## 3. Results

### 3.1. Sources of Fungal Particles

Information on the indoor storage of organic waste, indoor plants, and the presence of mouldy surfaces were extracted from 2014 questionnaire and analysed. The majority of owners had a container for organic waste and about 93% of them had indoor plants. The habits of storing the organic waste were similar among owners in different dwellings. 40% of owners reported mould contaminated surfaces in their dwellings, from which 42% were naturally ventilated and 18% mechanically ventilated. A quarter of them reported more than one contaminated site per dwelling. The most frequently reported site of mould contamination was the owners’ bedrooms (39% of samples), followed by the bathrooms (29% of samples), common living areas and basements (10% each).

To estimate the contribution of outdoor fungi indoors, information on the window-opening frequency in the main bedrooms were collected. 50% of dwelling owners opened windows every day, 31% opened windows sometimes, while the remaining 19% never opened windows during the sampling season (heating season). While the window-opening frequency was independent from the level of urbanization (*p* = 0.28), it was strongly associated to the energy efficiency status of dwellings (*p* < 0.001) and ventilation type (*p* < 0.001). Thus, during the heating period, occupants of naturally ventilated dwellings tended to open windows more frequently than those of mechanically ventilated (68% of occupants of naturally ventilated dwellings opened windows every day and 31% sometimes, as opposed to 10% and 33% in mechanically ventilated dwellings, respectively). As 93% of renovated (PB) dwellings were not mechanically ventilated, occupants in those dwellings tended also to open windows on a much more frequent basis than those in recently built Minergie homes that were all mechanically ventilated.

### 3.2. Cultivable Fungi in Settled-Dust

**Figure 2:**
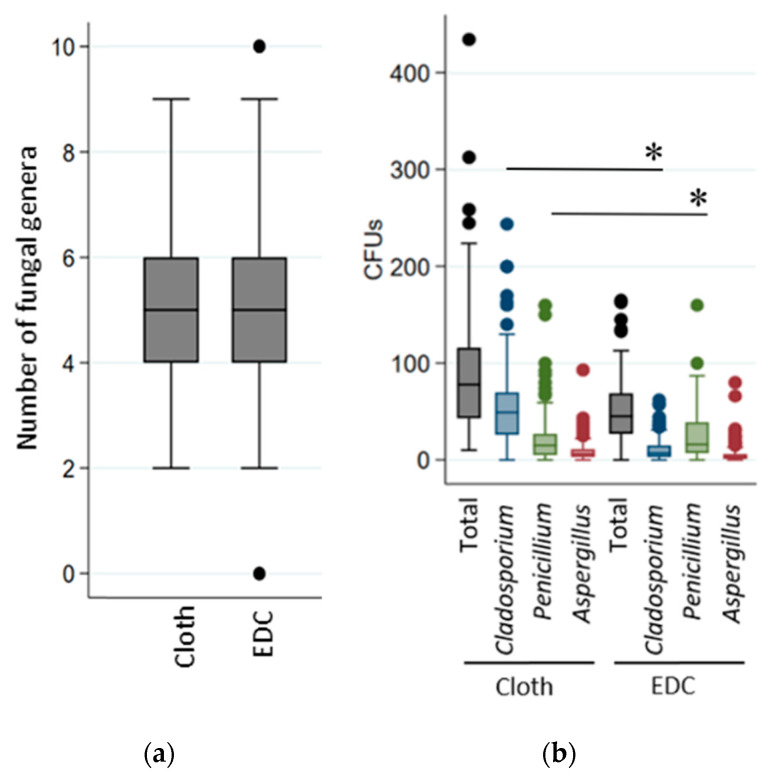
Comparison of the two sampling methods in relation to (**a**) the number of fungal genera detected; and (**b**) the CFUs number observed in total or for each of the three most abundant genera. The significant differences were indicated by * (*p* < 0.05).

To screen the overall fungal contaminants in owner bedroom, the settled dust was collected with a cloth and an EDC in order to determine the cultivable fraction. The two sampling methods yielded a similar diversity of genera in samples (*p* = 0.16; Figure 2a) and in the number of total CFUs (*p* = 0.85; Figure 2b). However, a higher number of *Cladosporium* CFUs—The dominant genus in samples—Was observed on cloth than on EDC samples (*p* = 0.03; Figure 2b). *Penicillium* and *Aspergillus* were found to be dominant in 22% of recent and 23% of retrofitted dwellings (they represented more than 70% of CFUs). Consequently, we specifically analysed the abundance of these genera in overall samples. We found that *Penicillium* was more abundant in the samples collected with the EDCs (*p* = 0.03; Figure 2b), which was independent of the dwelling location. No significant difference was observed in *Aspergillus* sampling between the two methods (*p* = 0.23; Figure 2b).

The number of *Penicillium* CFUs was lower in mechanically ventilated than in the naturally ventilated bedrooms when collected by the EDC method (*p* = 0.01; Table 1). Minergie dwellings had a lower accumulation of *Penicillium* and *Aspergillus* in the settled dust collected by the EDC method (Table 1). An increase in the number of *Cladosporium* (*p* = 0.03) and *Aspergillus* (*p* = 0.05) CFUs correlated with window-open frequency.

No significant difference in CFUs levels—Total or specific genus—Was observed among rural, peri-urban, and urban dwellings with both sampling methods. The *Cladosporium* CFUs level was higher in the bedrooms of dog owners in peri-urban environments (*p* = 0.05). Houses without growing moulds on any surface, exhibited a lower total number of CFUs in mechanically ventilated dwellings than in the naturally ventilated ones (*p* = 0.03).

The environmental determinant with the strongest association with the total number of CFUs in settled dust was the presence of a mouldy area in the same room (Table 2). However, a significant association was also observed with the detection of fungal growth anywhere in dwellings (Table 3). No significant difference in the total number of CFUs was observed between the contaminated dwellings with mechanical ventilation and those with natural ventilation, or between the recently built and the renovated dwellings.

### 3.3. Surface Mould Contamination in Dwellings

**Figure 3:**
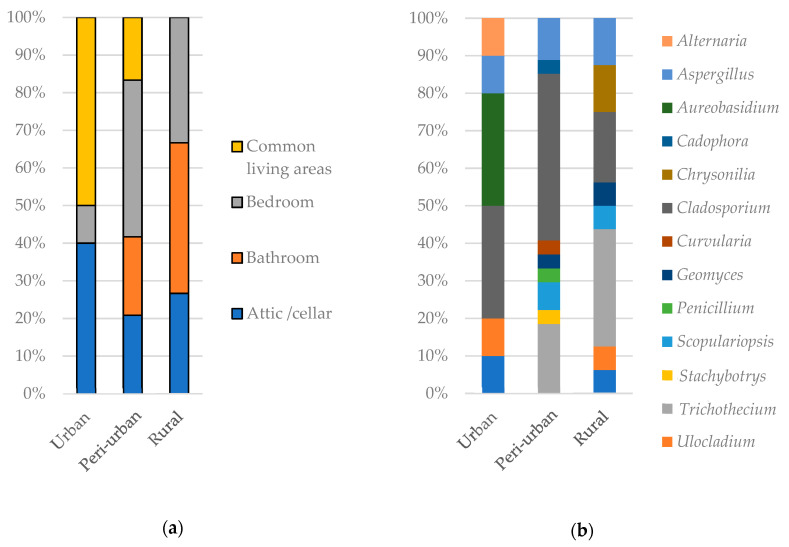
Fungal contamination of surfaces in dwellings from different locations: Urban (*n* = 19), peri-urban (*n* = 31) or rural (*n* = 29) depending on where it was observed in dwellings (**a**) and the percentage of fungi registered in relation to residency location (**b**).

Out of all dwellings in which occupants reported the presence of visible moulds in 2015–2016, 74% were confirmed in the lab to be contaminated by actively growing fungi. No difference in the proportion of houses contaminated by moulds was observed between mechanically and naturally ventilated dwellings as well as among urbanization categories. However, the location of mouldy surface in houses varied with the level of urbanization and it was independent from the ventilation system. In urban dwellings, fungal growth was mostly detected in common living areas and in attic/cellar spaces. In peri-urban dwellings, fungal growth was observed in bedrooms while in the rural dwellings, the fungal contaminant was predominantly found in bathrooms (Figure 3a).

Fungal diversity varied with dwelling location. A higher diversity of fungi growing on indoor surfaces was observed among peri-urban than among urban dwellings (Figure 3b). The most frequent fungal genera in overall samples were *Cladosporium*, *Trichothecium* and *Aspergillus* in 18, 10, and 6 samples, respectively. The most frequent fungal genera were *Aureobasidium* and *Cladosporium* in urban dwellings (in 30% of samples each), *Cladosporium* in peri-urban dwellings (44%) and *Trichothecium* in rural dwellings (31%) (Figure 3b).

**Figure 4:**
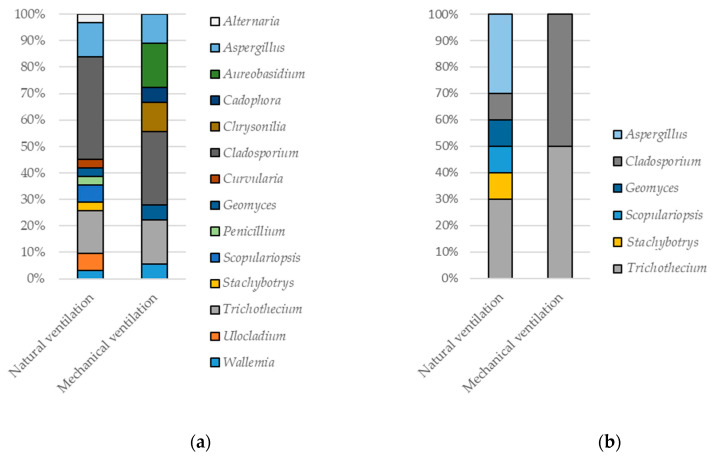
The fungal genera growing on surfaces in naturally and mechanically ventilated dwellings (**a**) in any living area (**b**) only in owners’ bedroom.

Interestingly, the diversity of growing fungi also differed substantially with the ventilation type (Figure 4). A higher fungal diversity was detected in naturally ventilated dwellings than those equipped with a mechanical ventilation (11 versus 8 taxa; Figure 4a). This difference was particularly prominent in owners’ bedroom (six versus two, Figure 4b). Five of those genera were detected in both types of dwellings (Figure 4). Very few genera growing on surfaces were also observed in settled dust (*Cladosporium, Aspergillus,* and *Penicillium*).

## 4. Discussion

This study generates data on fungal contamination and resulting occupant exposures in 149 energy efficient dwellings in Switzerland. The influencing variables included the level of urbanization (urban/rural), ventilation type (natural or mechanical) and dwelling specifications (recently built or renovated energy efficient dwellings). This dataset gives a realistic overview of the fungal contamination in the French-speaking part of Switzerland, a region characterized by a large diversity in the level of urbanisation.

Indoor fungal sources may modify fungal composition of settled dust. For two of them, namely the household bio-waste and potted house plants [16], the homogeneity of the sample population did not allow us to quantify their contribution to settled dust. In contrast, the presence of moulds in energy efficient dwellings was associated with a much higher number of total CFUs in settled dust. A similar result was previously reported in traditional houses [52,53]. Interestingly, the total number of CFUs in settled dust correlated with the presence of growing fungi anywhere in the house, but this correlation was much stronger when the fungi grew in the same room. This finding is compatible with the present knowledge on fungal particles behavior, including emission rates, dispersion distance and deposition, all which are influenced by fungal particle size (small particles will disperse further than bigger ones) [54]. Implications of fungal dispersion are discussed in previous studies describing a higher prevalence of health effects among owners of houses with visible moulds in the main living quarters, but not in other indoor areas [26,27,28].

The most common genera isolated in settled dust were *Cladosporium, Penicillium,* and *Aspergillus* Their presence in indoor environments was commonly reported in a large number of studies [55,56], in particular in traditional dwellings bordering a French area [57]. As a high number of spores per unit area (e.g., 10^2^ to 10^3^ CFU/cm^2^) can become aerosolized from natural building materials in homes [58], residents who sleep near a mouldy area can be exposed to relatively high concentrations of fungal particles. The situation is exacerbated when there is no sufficient ventilation indoors. Therefore, frequent opening of windows or adequate mechanical ventilation are necessary to prevent fungal propagules accumulation. While natural ventilation acts as a source of outdoor fungal particles, mechanical ventilation is expected to decrease the number of fungal particles of outdoor origin in indoor air. The presence of a mechanical ventilation, in particular in Minergie labeled dwellings, is strongly correlated with a lower number of total CFUs in settled dust, including *Penicillium* CFUs and *Aspergillus* CFUs. Despite the reduced diversity of fungal taxa, our results also suggest that mechanical ventilation is more effective than natural ventilation in removing some fungi. The slight difference between new and renovated dwellings could be caused by the mechanical ventilation system of new Minergie homes, which was integrated into the building design rather than added during the renovation. While mechanically ventilated non-contaminated dwellings had a lower number of fungal particles in settled dust, this was not the case in contaminated dwellings. We hypothesize that mechanical ventilation limits fungal infiltration from outdoor owing to particle arrestance on media filters, but does not perform better than the natural ventilation in terms of decreasing the number of fungal particles emitted by indoor mouldy areas. Measurements of the air exchange rate should be carried out in parallel with counting of CFUs to confirm these hypotheses.

The present study also suggests that mechanically ventilated dwellings had lower fungal diversity detected on surfaces, including indicator taxa of water-damage such as *Ulocladium* and *Stachybotrys*, compared to naturally ventilated ones. Related findings that the mechanical ventilation reduces the diversity of the microbiome have been reported for built environment and health-care facilities [59,60]. One implication of using mechanical ventilation is that it decreases the relative humidity in the bedrooms. The median relative humidity in bedrooms of dwellings with mechanical ventilation systems was previously reported to be 40%, while the levels in naturally ventilated dwellings was 50% [61]. To grow, a majority of moulds require high levels of moisture. A slightly lower accumulation of moisture can make a difference in the fungal taxa which may develop on indoor surfaces. Moreover, condensation can still accumulate in retrofitted energy efficient dwellings with natural ventilation at levels high enough to favour surface growth of at least *Aspergillus*, *Penicillium*, and *Cladosporium* [39]. In the present study, such a scenario may be an explanation for the difference observed in the diversity of moulds between mechanically and naturally ventilated dwellings.

Another notable result from this work is a potential importance of outdoor environment, i.e., rural, peri-urban, or urban, in influencing the diversity and location of fungi growing within dwellings. A higher diversity of fungi growing on indoor surfaces was observed among dwelling situated in peri-urban than in urban environments. Similar results have previously been observed between French dwellings with different levels of urbanization [62]. It is reasonable to assume that the diversity of outdoor fungi differs across rural, peri-urban, and urban environments, and that these differences are reflected in indoor fungi. Correlations between outdoor and indoor fungal community compositions were frequently identified [16]. Thus, factors governing the diversity of outdoor fungi, such as climatic and geographic position [63,64] and farming activities [16,65], indirectly impact indoor communities and consequently, fungal growth on indoor surfaces. Nevertheless, the infiltration rate of outdoor fungi indoors mainly depends on the outdoor conditions [41,66] and on the window-opening frequency. Thus, during the heating period, the contribution of outdoor fungi to indoor settled dust is expected to be lower [52]. Consequently, the impact of urbanization levels on the fungal content of indoor settled dust is less detectable, as similarly observed by Reboux et al. [56]. These previous findings may explain the observed difference in the diversity and richness of moulds between rural, peri-urban, and rural houses, but not of fungal particles detected in bedroom settled dust. However, a significant change in the number of *Cladosporium* CFUs has been associated to the frequency of window opening. Further research is needed to elucidate causes for different localizations in fungal growth as a function of urbanization levels.

In the present study, we identified the characteristics of energy-efficient dwellings that influence indoor microbial growth. Nonetheless, further research should be conducted to determine if the level of fungal contamination is high enough to affect inhabitants’ health. In such epidemiological studies, the isolation of thermophilic fungi such as *Aspergillus fumigatus* will be of interest.

## 5. Conclusions

The results of this study suggest that mechanically ventilated dwellings are more effective in preventing the infiltration of outdoor fungal particles, compared to naturally ventilated dwellings. Our findings support the need to further investigate the role that ventilation systems play in energy efficient dwellings with regard to control of moulds proliferation indoors and to maintain a number of airborne fungal particles low enough to protect occupants’ health. Particular attention has to be paid in dwellings situated in urban and peri-urban locations in which moulds were frequently reported in common living areas and bedrooms, respectively. This recommendation also includes newly constructed energy efficient dwellings. Counting fungal particles in settled dust provides an efficient detection tool of fungal growth in the same enclosed space. Whether the levels of fungal particles in energy efficient dwellings with or without mechanical ventilation have an impact on occupants’ health deserves to be investigated through large-scale cross-sectional field studies. A multidisciplinary approach involving both housing and public health providers is required to address this issue.

## Figures and Tables

**Table 1:** Average CFUs ± Standard Deviation of dominant fungal genera in settled dust of owners’ bedrooms.

	Type of Energy Efficient Dwellings		*p* Value	Ventilation Type		*p* Value
	Minergie (*n* = 44)	Renovated (*n* = 105)		Mechanical (*n* = 49)	Natural (*n* = 98)	
**Cloth sampling**						
*Cladosporium*	57 ± 54	54 ± 40	0.723	59 ± 53	54 ± 40	0.998
*Penicillium*	21 ± 31	21 ± 26	0.505	25 ± 37	19 ± 21	0.879
*Aspergillus*	12 ± 17	11 ± 25	0.566	12 ± 16	11 ± 25	0.492
EDC * sampling						
*Cladosporium*	9 ± 10	13 ± 14	0.065	9 ± 10	13 ± 14	0.053
*Penicillium*	19 ± 30	27 ± 23	**0.033**	21 ± 30	27 ± 23	**0.010**
*Aspergillus*	5 ± 11	8 ± 13	**0.004**	6 ± 11	8 ± 13	0.127

* EDC: electrostatic dust fall collector. Significant *p* values (<0.05) are in bold.

**Table 2:** Total average number of CFUs ± Standard Deviation identified in owners’ bedrooms with or without growing moulds depending on the energy efficient dwellings category and the method of settled dust sampling.

	Total CFUs ± SD in Owner Bedroom with		*p* Value
	No Fungal Growth	Fungal Growth	
**Settled dust sampled with cloth**			
All dwellings (*n* = 27)	51 ± 37	110 ± 67	**0.007**
Recent (*n* = 6)	18 ± 10	85 ± 42	0.054
Renovated (*n* = 21)	59 ± 37	119 ± 74	**0.022**
**Settled dust sampled with EDC**			
All dwellings (*n* = 27)	47 ± 29	45 ± 22	0.853
Recent (*n* = 6)	33 ± 34	57 ± 20	0.346
Renovated (*n* = 21)	50 ± 29	41 ± 22	0.423

Significant *p* values (<0.05) are in bold.

**Table 3:** Total average number of CFUs ± Standard Deviation identified in at least one location with or without fungal growth, depending on the energy efficient dwellings category and the method of settled dust sampling.

	Total CFUs ± SD in Dwellings with		*p* Value
	No Fungal Growth	Fungal Growth	
**Settled dust sampled with cloth**			
All dwellings (*n* = 60)	64 ± 44	100 ± 66	**0.034**
New (*n* = 19)	25 ± 15	101 ± 68	**0.044**
Renovated (*n* = 41)	74 ± 44	99 ± 65	0.191
**Settled dust sampled with EDC**			
All dwellings (*n* = 60)	42 ± 32	52 ± 30	0.243
New (*n* = 19)	53 ± 49	52 ± 26	0.937
Renovated (*n* = 41)	40 ± 28	53 ± 32	0.194

Significant *p* values (<0.05) are in bold.
